# EML2 and EML4 splice variants regulate microtubule remodeling during neuronal cell differentiation

**DOI:** 10.1016/j.jbc.2025.110252

**Published:** 2025-05-19

**Authors:** Venus Marasi, Rozita Adib, Laura O’Regan, Kees R. Straatman, Sally A. Prigent, Andrew M. Fry

**Affiliations:** 1 Department of Molecular and Cell Biology, University of Leicester, Leicester, UK; 2 Advanced Imaging Facility, Core Biotechnology Services, University of Leicester, Leicester, UK

**Keywords:** differentiation, EML2, EML4, alternative splicing, microtubules, overexpression, depletion, neurons, microscopic imaging

## Abstract

Neurons depend on microtubule organization for axon and dendrite formation during differentiation. Yet, our understanding of how microtubules are remodeled during this process is far from complete. Echinoderm microtubule-associated protein-like (EML) is a family of microtubule-associated proteins (MAPs) highly expressed in neuronal cells. Database analysis revealed that EMLs are subject to alternative splicing in their N-terminal regions, leading to the production of one long (L) variant containing a complete N-terminus and TAPE domain, and possibly several short (S) variants with a truncated N-terminal region. We investigated EML4 and EML2 splice variants during SH-SY5Y neuronal cell differentiation, examining their expression, localization, and effects on neurite outgrowth and branching. We found that differentiation led to decreased expression of the EML4-L but not the -S variant. Moreover, expression of the EML2-L variant increased, while EML2-S expression decreased. Overexpression of EML2-L and EML2-S led to increased and decreased neurite lengths, respectively. Interestingly, neurite branching increased in EML2-S-transfected cells. Using immunofluorescence microscopy, we found that EML2-L and EML4-L but not EML2-S and EML4-S localized strongly to interphase microtubules regardless of whether cells had differentiated or not. Depletion of both EML4 and EML2 led to neurite outgrowth. We propose that the presence of the N-terminal microtubule-binding region in the long variants of EML2 and EML4 promotes stabilization of microtubules and neurite extension, while short variants favor branching. Together, these data suggest that the regulated expression and localization of different splice variants of EML proteins are crucial to microtubule remodeling during neuronal differentiation.

Differentiation is a crucial step in the wiring of the neuronal network that highly depends on cytoskeleton-based processes (1). *In vitro*, undifferentiated neuronal cells continuously divide and grow in clusters that may stack rounded cells on top of one another. The proliferation rate is reduced upon differentiation, and cells stop forming clusters as differentiated cells exit the cell cycle. Differentiated cells adopt a pyramidal shape as neurites extend (2, 3, 4). Neurite outgrowth is triggered by actin polymerization and stable microtubules, forming small bundles of cytoplasmic microtubules that extend in various directions and later develop into axons and dendrites that can grow to hundreds of micrometres in length (1, 3, 5, 6). Axons and dendrites differ structurally due to their distinct microtubule polarity. Axons have uniformly plus-end-out microtubules, while dendrites exhibit mixed polarity, with both plus and minus ends capable of facing away from the cell body (4, 7).

Echinoderm microtubule-associated protein-like (EML) is a family of microtubule-associated proteins (MAPs) that regulate the assembly and stability of microtubules (8, 9). Overall, humans express six EML proteins, with EML1, 2, 3, and four sharing similar structures, consisting of an N-terminal domain comprising a trimerization domain (TD) and basic region, and a C-terminal domain comprising conserved WD40 repeats (repeats of tryptophan-aspartate) and a conserved HELP motif (10). A coiled-coil trimerization domain contains an amphipathic α-helix with conserved hydrophobic residues, predominantly leucine and valine, facilitating EML trimerization through hydrophobic interactions and side-bridge stabilization. This enhances EML binding to the microtubule lattice (11, 12). Microtubule lattices are negatively charged due to acidic glutamate-rich E-hooks on α- and β-tubulin tails. A basic N-terminal region in EMLs interacts electrostatically with these E-hooks (12, 13, 14). X-ray crystallography shows that WD repeats form two tandem β-propellers connected at a ∼50° angle, creating the tandem atypical propeller in EML (TAPE) domain, which binds soluble α/β tubulin heterodimers *via* its concave surface (15, 16). The HELP motif, a conserved ∼60 amino acid region, links the two propellers (15).

Some findings provide strong evidence for the role of EML proteins in regulating cytoskeletal microtubules during neuronal development. For instance, in neuronal progenitors, EML1 is found at the spindle poles during metaphase and the equatorial region during anaphase and telophase (17). A point mutation in the EML1-TAPE domain causes neuronal heterotopia in both humans and rodents, in which immature neurons are not able to migrate properly during the early stage of brain development (18). The high expression of EML4 in neurons raises the prospect that this protein, like Tau, may have a role in stabilizing neuronal microtubules, and as a trimer, EML4 could bind to more than one tubulin dimer in the microtubule lattice (10). In contrast, earlier studies in HeLa cervical cancer cells suggest that EML2 short variant (ELP70) binds tubulin *in vitro via* its HELP motif, inhibiting seeded nucleation and increasing microtubule catastrophes by weakening lateral interactions between protofilaments (19). Other findings, however, show that EML2-S tracks shrinking microtubule plus ends, impacting dynamics to promote stabilization. The author suggests that the TAPE domain in EML2-S contains a recognition motif for the C-terminal tail of tyrosinated α-tubulin (20).

A study on gene expression during neuronal differentiation in mouse hippocampal neurons *in vitro* analyzed 20,398 genes, including 1456 microtubule cytoskeleton-associated genes, using microarray analysis (21). According to this study, of the six EMLs, only EML2 mRNA increased, while EML4 was downregulated and other EMLs remained quite constant, suggesting EML2's greater role in differentiation. The study did not explore splice variants, although alternative splicing alters EML sequences as seen in an EML4 short variant lacking 60 amino acids in its N-terminal microtubule-binding domain (22). Similarly, HuEMAP-2 (EML2) transcripts undergo splicing and in humans, EML2's N-terminal trimerization domain is completely absent from the short variant (20, 23). Moreover, EML5 which is highly expressed in post-mitotic neurons exists as two splice variants, EML5^a^ and EML5^b^, which encode C-terminal truncations of EML5 (24).

Alternative splicing of EML2 and EML4 leads to the formation of one ‘long’ variant, known as a full-length variant, that contains a complete N-terminus as well as the TAPE domain, and the production of several possible ‘short’ variants that lack different parts of their N-terminus. This, together with preliminary data generated in our lab, has led us to propose that longer EML protein variants that contain the N-terminal microtubule-binding region may promote microtubule stabilization, whereas shorter variants that lack this region may promote microtubule destabilization. Finally, we hypothesize that a switch in alternative splicing of EML proteins could have major consequences on microtubule organization, which is important for neuronal cell differentiation. It should be noted that although there are additional splice variants of EML2 and EML4, in this study, we have chosen to concentrate on one long and one short variant of these proteins.

Overall, our data suggest that morphological changes in SH-SY5Y cells appear to be influenced by expression of the EML2 and EML4 splice variants during differentiation. We also found that the long variants of EML4 and EML2, which have both an N-terminal domain that associates with polymerized microtubules and a C-terminal TAPE domain that binds soluble tubulin heterodimers, promote the stabilization of microtubules and neurite extension. Since EML4 short contains a portion of the microtubule-binding domain, it did not significantly change the morphology of neuronal cells. However, the short variant of EML2 that lacks the full N-terminal microtubule-binding domain rather appears to cause neurite shortening. Interestingly, the shortening of neurites seems to be accompanied by an increase in the nucleation of neurites and neurite branching.

## Results

### Potential splice variants of human EML2 and EML4

It has previously been demonstrated that mouse EML4 is subject to alternative splicing during embryonic development and that different EML4 splice variants are expressed in varying quantities in various mouse brain regions (10). Interestingly, the mouse brain regions analyzed did not express exon two of EML4, which encodes a portion of the N-terminal domain. Additionally, recent evidence revealed that EML2 also undergoes splicing in humans to generate a short version that entirely lacks the N-terminal trimerization domain (20).

**Figure 1:**
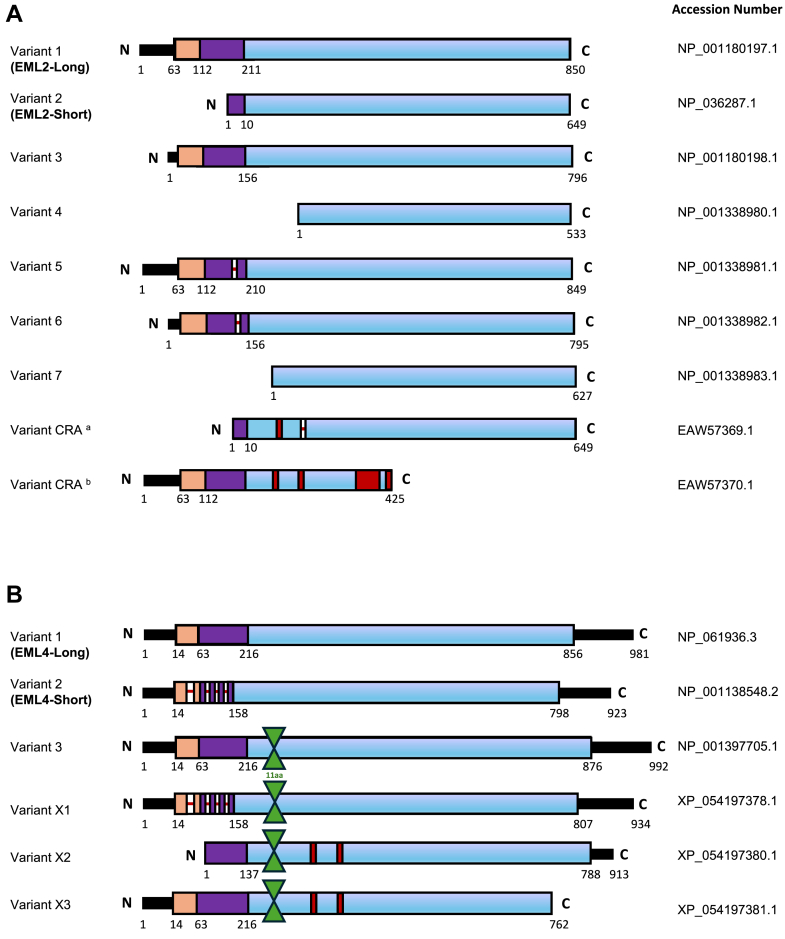
**Schematic representation of the potential splice variants of human EML2 and EML4.***A*, EML2 splice variants. The EML2 full length (variant 1) is made up of 22 exons and 850 amino acids (aa) consisting of a trimerization domain (TD) (*orange*) and a basic area (*purple*) in the N-terminus, and a TAPE domain (*blue*) in the C-terminus. EML2-Short (variant 2) lacks exons one to four and consists of 649 aa. Exon 1, which contains 57 aa, is absent from variant 3. Variant four lacks 317 aa from exons one through 7 as well as a portion of exon 8. Residue 172 is absent from variant 5 (*red line*), and exon one and residue 172 are absent from variant 6. Variant seven lacks 223 aa, exons one through five and a portion of exon 6. Variant CRA^a^ contains 649 aa. This protein lacks 207 aa from the N-terminus, residue 234 is mismatched (*red*) and the residue 388 is missing. Variant CRA^b^ contains the full portion of the N-terminus. In this protein residues between 234–388 and also 403–419 are mismatched and the rest of the C-terminus is missing. Accession numbers are indicated on the *right*. *B*, EML4 splice variants. EML4 full-length (variant 1) is comprised of 23 exons and 981 aa including a trimerization domain (TD) (*orange*) and a subsequent basic region (*purple*) in the N-terminus and the TAPE domain (*blue*) in the C-terminal region. EML4-Short (variant 2) is made up of 923 aa. 58 aa missed out from the N-terminus (*red lines*). An additional 11 aa are present in variant three between residues 222 and 223 (*green*). Variant X1 is the combination of variants 2 and 3. The N-terminus lacks 58 aa, while there are an extra 11 aa between residues 222 and 223. In variant X2 the first 79 aa from N-terminal are missing. There are an extra 11 aa between residues 222 and 223 and also residues 282 and 382 are mismatched (*red*). Variant X3 contains an extra 11 aa between residues 222 and 223 and also residues 282 and 382 are mismatched. Accession numbers are indicated on the *right*.

To identify potential alternative human transcripts of EML2 and EML4, we searched the NCBI Genebank database. Nine possible variants were identified for EML2 and six for EML4 (Fig. 1). Of the nine EML2 variants, variant one encodes the full-length protein, containing 22 exons. It comprises 850 amino acids including a trimerization domain (TD) and a basic region at the N-terminus, and a TAPE domain in the C-terminus. Variant 2 consists of 649 amino acids and lacks exons 1 to 4 (Fig. 1*A*). Of the six EML4 variants, variant 1, a full-length protein, contains 23 exons and is made up of 981 amino acids including a trimerization domain (TD) and a basic region at the N-terminus, and a TAPE domain in the C-terminus. Variant 2 consists of 923 amino acids. In this protein, 58 amino acids missed out from regions between residues 23 and 45, 68 and 77, 122 and 132, and 146 and 167 in Exons 2, 3, and 4 (Fig. 1*B*).

In this study, variants 1 and 2 of EML2 and EML4 were selected as they differ in a portion of the N-terminal domain required for microtubule binding. These are subsequently referred to as EML2-L and EML2-S and EML4-L and EML4-S, respectively. We hypothesized that the different capacities of these proteins to bind microtubules as a result of expression of their truncated N-terminal regions could explain the distinct functions they perform during neuronal differentiation.

### Differentiation of SH-SY5Y cells leads to altered expression of EML2 and EML4 splice variants

It is unknown how EMLs function in post-mitotic neurons and during neuronal development. Evidence suggests that various EML proteins may either stabilize or destabilize microtubules, and this could be significant for cell differentiation, migration, and division (23). For instance, EML4 is known as a microtubule stabilizer while EML2 acts as a microtubule destabilizer in cultured cells. Specifically, EML2 binds to tubulin and prevents microtubule development and nucleation, shortening microtubules in the process (19). We therefore wanted to determine whether we could detect changes in expression of the long and short variants of EML2 and EML4 during retinoic acid (RA)-induced differentiation of SH-SY5Y neuronal cells.

**Figure 2:**
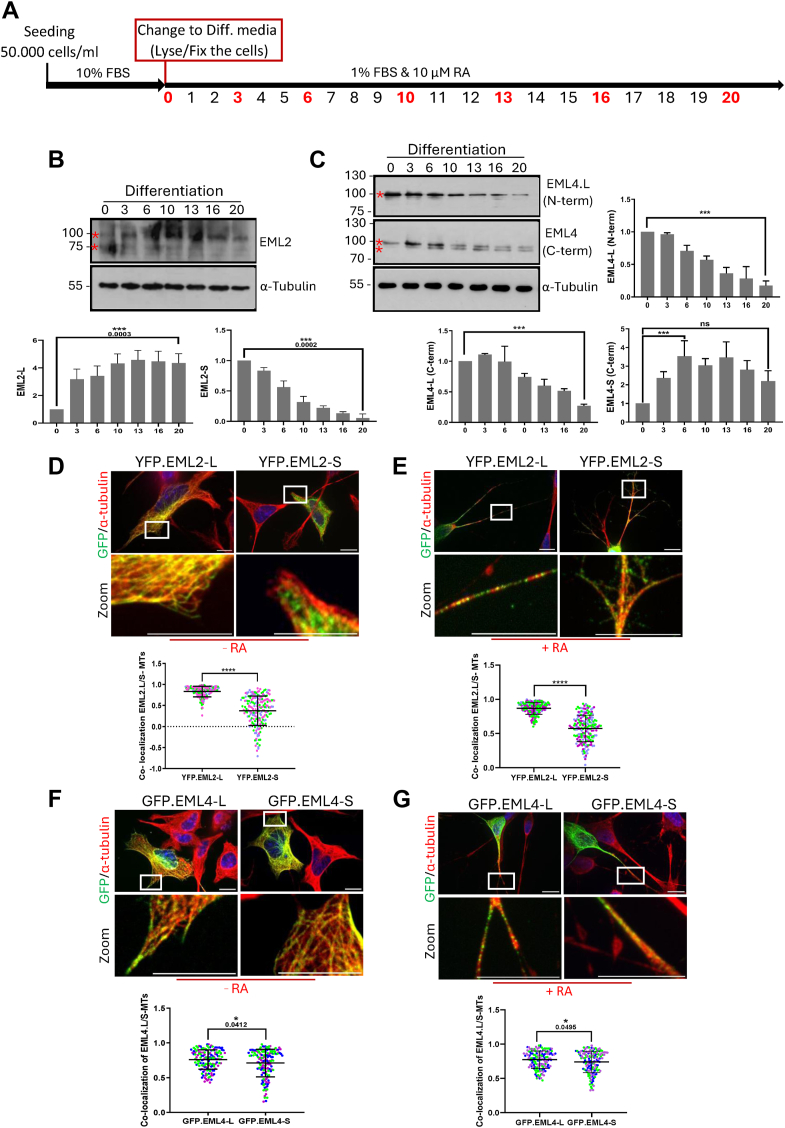
**Differentiation of SH-SY5Y cells alters the expression of EML2 and EML4 splice variants, with the long variants showing stronger co-localization with microtubules compared to the short variants.***A*, the timeline of a method for differentiating SH-SY5Y cells. SH-SY5Y cells were cultured in a medium containing 10% FBS. After the cells reached 60% confluency (Day 0), the medium was replaced with fresh medium containing 1% FBS, and 10 μM Retinoic acid (RA) was added dropwise to cells. Red numbers indicate days on which cells were collected for Western blot analysis or fixed for immunostaining. Furthermore, the culture medium and RA were refreshed in the plates on the remaining days. *B* and *C*, lysates were prepared from SH-SY5Y human neuronal cells at the indicated days (D0 to D20) after the addition of RA to induce differentiation. Western Blots were undertaken with EML2, EML4 (N-term), and EML4 (C-term) antibodies. The α-tubulin antibody was used as a loading control. Molecular weights (kDa) are indicated on the *left*. Asterisks indicate quantified bands. Bar charts represent the intensity of EML2-L, EML2-S, EML4-L and EML4-S across the 20 days of induction with RA, normalized to α-tubulin. The EML4 bar chart on top shows the result with the EML4 N-terminal antibody, while the bottom ones show the result with the EML4 C-terminal antibody of both the long (EML4-L) and short (EML4-S) variants. Data obtained from three repeats, n = 3 (±S.D.). ∗∗∗ indicates a *p*-value of < 0.001, and ns (not significant) indicates a *p*-value of 0.1652. *D* and *E*, SH-SY5Y cells were fixed 24 h after transfection with EML2-L and -S variants (−RA) and 72 h after RA treatment following the transfection (+RA). Cells were stained with α-tubulin (*red*) and GFP (*green*) antibodies. DNA was stained with Hoechst 33258 (*blue* in merge); Magnified zoomed views are shown from the boxed areas. Scale = 10 μm. The mean Pearson's correlation coefficient for co-localization between EML2 variants and microtubules (MTs) in undifferentiated and differentiated SH-SY5Y cells was calculated from five measurements of 10 cells per experiment. n = 3 (±S.D.). *p*-value < 0.0001. Multiple coloured dots are the indication of different repeats. *F* and *G*, SH-SY5Y cells transfected with EML4-L and -S variants were fixed before (−RA) and after 72 h RA treatment (+R). Cells were then stained with α-tubulin (*red*) and GFP (*green*) antibodies. DNA was stained with Hoechst 33258 (*blue* in merge). Magnified zoomed views are shown from the box areas. Scale = 10 μm. The mean Pearson's correlation coefficient for co-localization between EML4 variants and microtubules (MTs) in undifferentiated and differentiated cells was calculated from five measurements per cell from three independent experiments. The experiment was repeated at least three times (±S.D.). ∗ *p*-value < 0.05.

SH-SY5Y cells were grown in the medium containing 1% FBS and 10 μM RA. Cell lysates were prepared from cells collected on Days 0, 3, 6, 10, 13, 16, and 20 of RA treatment and subjected to Western blot analysis using antibodies against EML2 and EML4 (Fig. 2*A*). Differentiated SH-SY5Y cells exhibited strikingly longer neurites compared to undifferentiated cells; hence, neurite outgrowth served as a differentiation marker (Fig. S1, *A* and *B*). Since the N-terminus is largely absent in the short variant of EML2, an antibody raised against sequences within the protein's C-terminus that can detect both long and short variants was used. The molecular weight of EML2-L is 92 kDa while EML2-S has a molecular weight of 71 kDa. Our data showed that EML2-L expression increased within 20 days of differentiation with RA, with a noticeable increase after Day 10 of differentiation. By contrast, a decrease in EML2-S protein expression was observed (Fig. 2*B*).

Acetylation and detyrosination are markers of microtubule stabilization in highly differentiated cells such as neurons. Detyrosination is also associated with neurite initiation (25, 26). In addition, EML2-S was shown to bind to tyrosinated tubulin while EML2-L can bind equally to tyrosinated and detyrosinated tubulins (20). Thus, we also analyzed the effect of differentiation on tubulin modifications by Western blotting with antibodies against acetylated and detyrosinated tubulin. Surprisingly, the level of expression of both detyrosinated and acetylated tubulin remained unchanged throughout the entire differentiation process (Fig. S2, *A* and *B*). This may be because distinct sections of the neuron, such as the cell body, dendrites, axons, and growth cone, require these tubulin post-translational modifications at different stages of development.

To assess the effect of differentiation on EML4 expression, two commercial EML4 antibodies were selected that could distinguish between long and short variants. An EML4 antibody raised against the C-terminus (EML4-CTD) should detect both the long and the short variants, while one raised against the N-terminus (EML4-NTD) would only detect the long variant. Western blotting with both antibodies demonstrated a 4-fold reduction in expression of the EML4-L variant. In addition to the 100 kDa EML4-L band, the EML4-CTD antibody also revealed a second smaller band with a molecular weight of 92 kDa which represents the EML4-S variant. This protein was virtually undetectable on Day 0 and expression increased during differentiation, reaching a plateau on Day 6 (Fig. 2*C*).

Taken together, our data suggest that as EML4-L stabilizes microtubules, its downregulation may lead to a rise in the instability of microtubules, which would then promote neurite outgrowth.

### EML2-L and EML4-L but not short variants co-localize strongly with microtubules in SH-SY5Y cells

To investigate whether the absence of the microtubule-binding domain from the N-terminus of short variants of EML2 and EML4 affects their localization to microtubules, plasmid constructs were generated containing YFP and GFP tags, respectively, at their N-terminus to examine the localization of the EML2 and EML4 variants. Following transfection, an immunofluorescence microscopy of cells that had been transfected with long and short EML2 constructs demonstrated that EML2-L showed markedly higher co-localization with microtubules than EML2-S (R = 0.9 vs R = 0.4). This is consistent with the presence of the N-terminal microtubule-binding domain in EML2-L but not in EML2-S (Fig. 2*D*), suggesting that EML2-S has a reduced capacity to associate with microtubules. Induction of differentiation by treatment with RA resulted in the same co-localization pattern, where EML2-L exhibited higher microtubule co-localization than EML2-S (Fig. 2*E*).

Analysis of localization of the GFP-EML4 long and short variants demonstrated that while EML4-S was highly localized to microtubules (R = 0.72), EML4-L exhibited even higher microtubule co-localization (R = 0.87) compared to EML4-S (Fig. 2*F*). These results are consistent with previous studies showing that EML proteins bind to microtubules *via* their N-terminal region (12). Although EML4-S lacks a portion of the microtubule-binding domain at the N-terminus, it still has some capacity to bind microtubules, likely because it still retains the key residues required for microtubule binding. The co-localization of both EML4-L and EML4-S remained unchanged following RA treatment (Fig. 2*G*).

### EML2-L and -S have opposing effects on SH-SY5Y neurite length and branching

**Figure 3:**
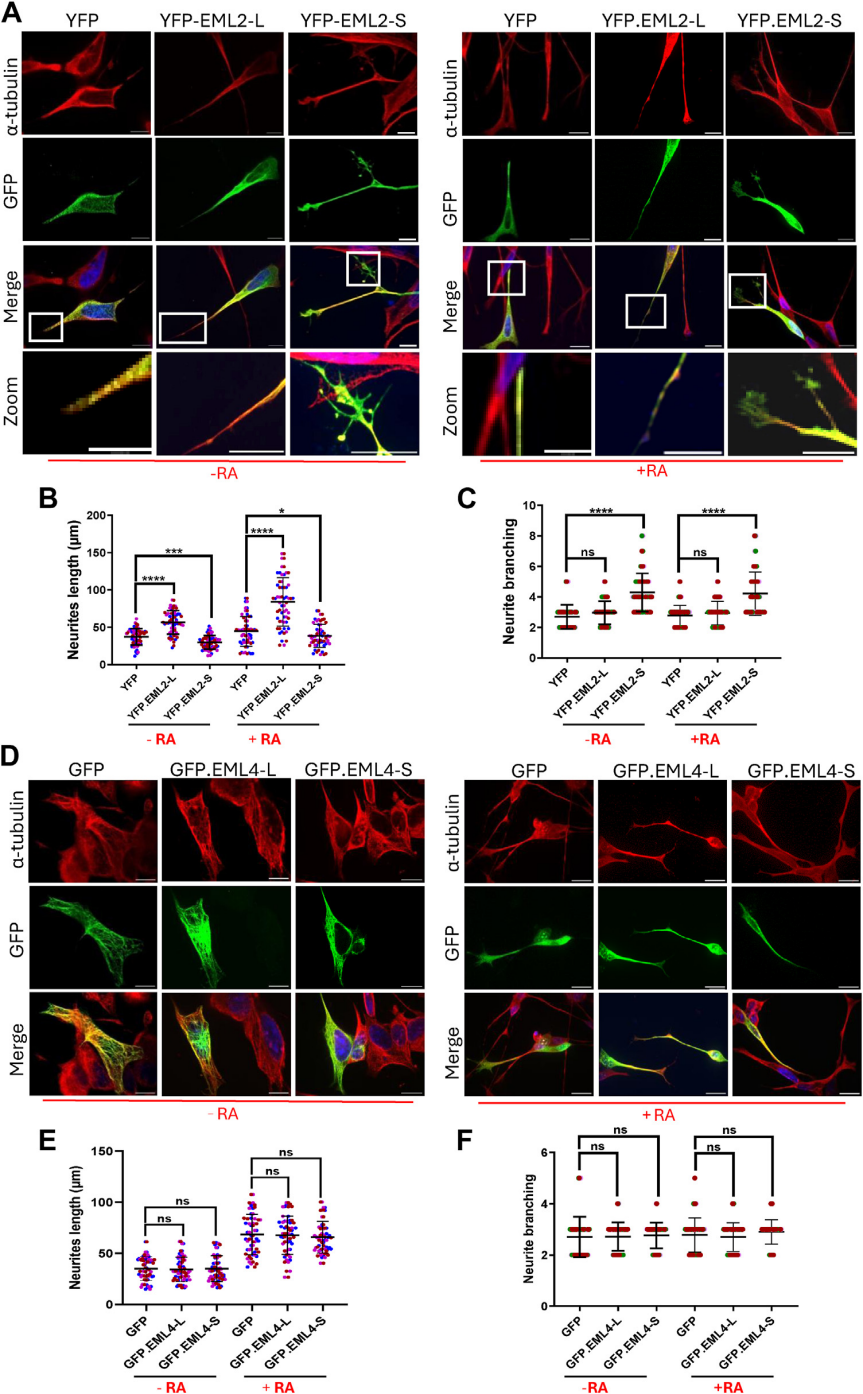
**Overexpression of EML2-L and -S leads to an increase in neurite length and branching respectively before/after SH-SY5Y differentiation while overexpression of EML4-L and -S does not alter cell morphology.***A*, SH-SY5Y cells were fixed 24 h after transfection with YFP only plasmid, YFP-EML2-L and -S variants and 72 h after RA treatment following the transfection. Cells were then stained with α-tubulin (*red*) and GFP (*green*) antibodies. Merged images include DNA stained with Hoechst 33258 (*blue* in merge). Zoom images indicate neurite branching in transfected cells. Scale = 10 μm. *B*, dot plots indicate the length of neurites measured from the nucleus to the tip of the longest neurite in each transfected cell before and after treatment with RA. In this study, negative controls are cells transfected with YFP alone. The experiment was performed at least three times and the length of neurites in 20 cells counted in each experiment (±S.D.). ∗*p* < 0.05, ∗∗∗∗ *p*-value < 0.0001. Multiple coloured dots are the indication of different repeats. *C*, the dot-plot indicates the number of protrusions branched from each individual neurite in SH-SY5Y cells transfected with YFP alone or the EML2-L or -S variants after 24 h. This experiment was done at least three times and the neurite branching in 20 cells counted in each repeat (±S.D.). ∗∗∗∗ *p*-value < 0.0001, ns: not significant. *D*, SH-SY5Y cells were fixed 48 h after transfection with GFP only plasmid. GFP-EML4-L and -S variants and 72 h after RA treatment following the transfection. Cells were stained with α-tubulin (*red*) and GFP (*green*) antibodies. Merged images include DNA stained with Hoechst 33258 (*blue* in merge). *E*, dot-plot show the length of neurites in cells transfected with GFP alone, EML4-L and EML4-S with or without RA treatment. The experiment was done at least three times and the length of 20 cells was counted in each experiment (±S.D.). The *p*-value is indicated. *F*, the dot-plot indicates the number of protrusions branched from each individual neurite in SH-SY5Y cells transfected with GFP alone or the EML4-L or -S variants after 24 h. This experiment was done at least three times and the neurite branching in 20 cells counted in each repeat (±S.D.). ns, not significant.

Having established differences in the microtubule-binding properties of EML2 and EML4 long and short variants, we aimed to determine whether these changes result in altered SH-SY5Y cell morphology and neurite extension before and after RA-induced differentiation. Confocal microscopy analysis of SHSY-5Y cells that had been transfected with EML2 long or short variants and subsequently treated with RA revealed that 24h after transfection without RA induction, the average neurite length was higher in cells transfected with EML2-L than in cells transfected with YFP alone. By contrast, transfection with EML2-S led to a significant reduction in mean neurite length (Fig. 3, *A* and *B*).

As expected, differentiation led to an increase in overall neurite length in all cell groups. However, cells transfected with EML2-L showed longer neurites compared to the control cells. The mean neurite length of EML2-L transfected cells was 80 μm after RA-induction. Furthermore, EML2-S transfected cells displayed a shorter mean neurite length of 35 μm compared to controls, which had a mean neurite length of 44 μm. Overall, this demonstrates that different EML2 splice variants have opposing effects on neurite length in SH-SY5Y cells with or without differentiation. Although following differentiation, neurite length increased in all cases, cells transfected with EML2-S still exhibited shorter neurites compared to those transfected with EML2-L (Fig. 3, *A* and *B*).

We next measured whether expression of the different EML2 splice variants had any impact on neuronal cell branching in transfected cells with and without RA treatment. Intriguingly, SH-SY5Y cells transfected with YFP-EML2-S exhibited considerable morphological alterations as a result of neurite branching. After 24 h of transfection, cells transfected with EML2-S exhibited significantly more (*p* < 0.0001) branching than cells transfected with EML2-L or YFP alone. EML2-S-transfected cells had 3 to 8 branches per cell, while EML2-L or YFP alone transfected cells had 2 to 5. Consistent results were obtained following RA treatment (Fig. 3*C*). This result suggests that the diminished capacity of EML2-S to bind microtubules leads to the formation of branched-short neurites.

So far, our data revealed that differentiation did not affect the changes in neurite length induced by EML2 when RA was applied after transfection with YFP-EML2 constructs. To further understand EML2's role in differentiation, we next wished to determine whether the expression of EML2 would affect neurite length in cells that had already undergone RA-induced differentiation. To do this, SH-SY5Y cells were differentiated with RA for 96 h, before being transfected with YFP only, YFP.EML2-L, and -S. for a further 24 h (Fig. S3, *A* and *B*). Immunofluorescence microscopy revealed that there was no significant difference in neurite length between cells transfected with YFP.EML2-L and YFP-only (average neurite length of 68 and 70 μm, respectively). However, cells were transfected with YFP.EML2-S exhibited shorter neurites than control cells (average neurite length 40 μm) (Fig. S3*C*). Thus, overexpression of the EML2-S causes a reduction in the length of neurites in already differentiated SH-SY5Y cells.

Our previous data revealed that both EML4-L and EML4-S variants colocalize with microtubules but to a different extent. Therefore, we next examined whether overexpression of these proteins had any impact on neurites. To address this, SH-SY5Y cells were transfected in duplicate wells with constructs expressing GFP alone or GFP.EML4-L and -S for 24 h. One well from each pair was treated with RA for 72 h. Cells were then fixed and stained with antibodies against GFP and α-tubulin and the length of neurites was analyzed by confocal microscopy using Fiji (Fig. 3*D*). Our results showed that before RA-induction, there was no significant difference in the length of neurites between the control cells and cells transfected with EML4-L or EML4-S (*p* = 0.8209 and 0.9931, respectively). The length of neurites in all three sets was highly variable, with measurements between 15 μm and 62 μm, with a mean of 38 μm. The addition of RA increased the overall neurite length (range 28 μm to 110 μm, mean of 68 μm) in control and EML4-transfected cells to a similar extent. Analysis of neurite branching also demonstrated that there were no significant differences among the different populations, both before and after RA treatment (Fig. 3, *E* and *F*). This result suggests that EML4’s capacity to stabilize microtubules causes transfected cells to retain their morphology.

### Depletion of EML2-L increases neurite length and branching in undifferentiated SH-SY5Y cells

Our data demonstrate that overexpression of different EML2 splice variants in SH-SY5Y cells significantly affects neurite length and branching, providing exciting insights into how these MAPs might regulate neuronal cell differentiation. To further investigate the role of EML2 in the regulation of neurite extension and branching during neuronal cell differentiation, we examined the effects of EML depletion using siRNA. First, we design siRNAs that individually target the long and short splice variants of EML2. Considering exons one to four are missing in the EML2 short variant, an siRNA raised against this region should deplete the EML2 long variant without affecting the short variant. Thus, three siRNAs were used, siEML2.1, siEML2.2, and siEML2.3, that specifically depleted the EML2 long variant, and two siRNAs, siEML2.4 and siEML2.5, that deplete both EML2 short and long variants.

**Figure 4:**
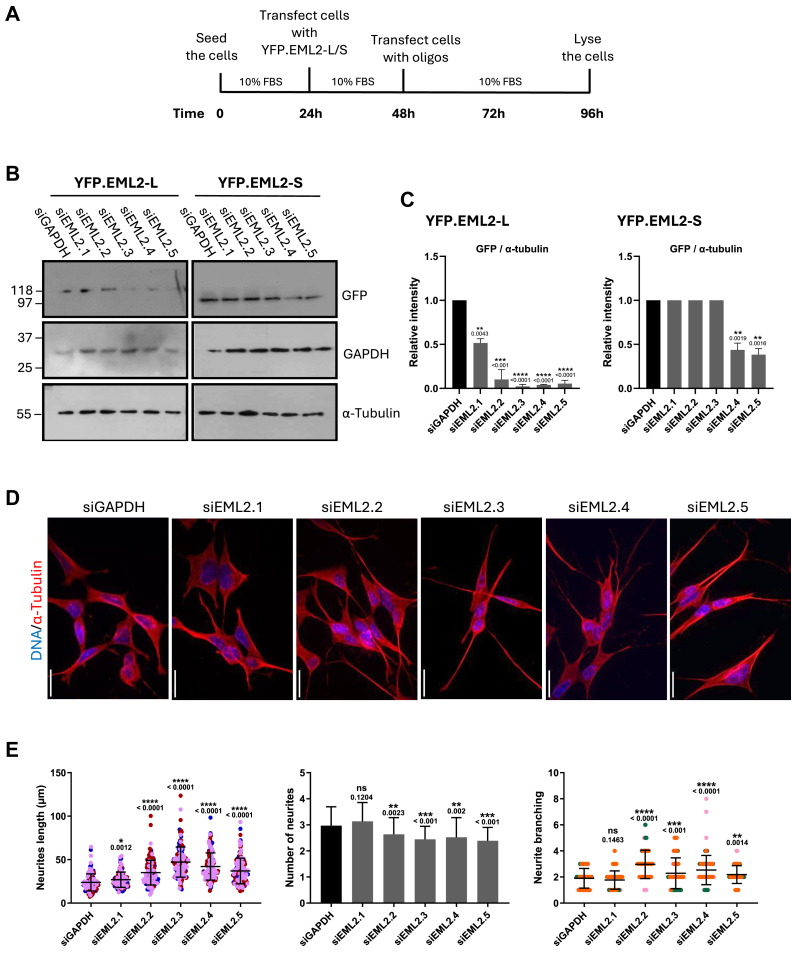
**Depleting EML2 splice variants increases neurite length and branching in undifferentiated SH-SY5Y cells.***A*, the timeline showing the process of overexpression and depletion of EML2-L and -S in SH-SY5Y cells. *B*, to optimize EML2-L and -S depletion in SH-SY5Y cells, cells were transfected with YFP-tagged EML2-L and -S variants for 24 h before being depleted with siEML2.1 to siEML2.5. Transfected cells were also depleted with siGAPDH as a control. Cells were analyzed by Western blot with the antibodies indicated. Molecular weights (kDa) are indicated. *C*, the bar charts represent the intensity of GFP normalized to α-tubulin, n = 3, (±S.D.). *p*-values are indicated. Cells depleted with siEML2.1 to 5 are compared to the control cells that are depleted with siGAPDH. *D*, SH-SY5Y cells were transfected with the EML2 siRNAs indicated for 48 h before analysis by immunofluorescence microscopy. Cells were stained with α-tubulin antibodies (*red*) and DNA with Hoechst 33258 (*blue* in merge); scale = 20 μm. *E*, neurite length was measured from the nucleus to the tip of the longest neurite in each individual cell as shown in (*A*). The dot plot reveals the length of neurites after EML2 depletion. The bar chart shows the number of neurites per cell based on cells shown in (*D*). The dot plot shows the number of neurite branches per cell based on cells shown in (*D*). This experiment was repeated at least three times, and 20 individual cells were measured in each repeat (±S.D.). *p*-values are indicated.

As there are no commercial EML2 antibodies available to allow reliable detection by Western blotting, SH-SY5Y cells were first transfected with the YFP-tagged EML2-L and -S before siRNA depletion of EML2, allowing Western blotting with GFP antibodies to be used to determine oligo efficiency (Fig. 4 *A* and *B*). Our results showed that all EML2 siRNAs were able to deplete the EML2-L variant without affecting GAPDH or α-tubulin. As expected, siEML2.4-siEML2.5 but not siEML2.1-siEML2.3 also led to a decrease in the EML2 short variant (Fig. 4*C*). Thus, we have identified siRNA oligos able to efficiently deplete EML2.

To test the consequences of EML2 depletion on neurite outgrowth, undifferentiated SH-SY5Y cells were treated with each individual EML2 siRNA oligo for 48 h before analysis by immunofluorescence microscopy with α-tubulin antibodies (Fig. 4*D*). Measurements of neurite lengths revealed a significant increase in neurite length with all five EML2 siRNAs. In support of this being a specific response to a loss of EML2, the change was smallest with siEML2.1, which was less effective at EML2 depletion. The mean neurite length of siEML2.1- siEML2.5 was as follows, respectively, 25 μm, 40 μm, 50 μm, 45 μm, and 40 μm (Fig. 4*E*). The number of neurites and neurite branching was also quantified after EML2 depletion. Interestingly, the number of neurites significantly decreased upon depletion of EML2 in four out of the five siRNAs, with only siEML2.1 showing no significant change. Meanwhile, there was also a significant increase in neurite branching upon EML2 depletion (Fig. 4*E*). As these results were observed to a similar extent with siRNAs that deplete only the long variant of EML2 as compared to both variants, we conclude that these phenotypes are primarily a consequence of depletion of the EML2-L. This result suggests that depleting EML2 long and short mimics some of the RA-induced processes where the neurite length increases, and the number of neurites decreases.

### Depleting EML4 splice variants increases neurite length and branching in undifferentiated SH-SY5Y cells

**Figure 5:**
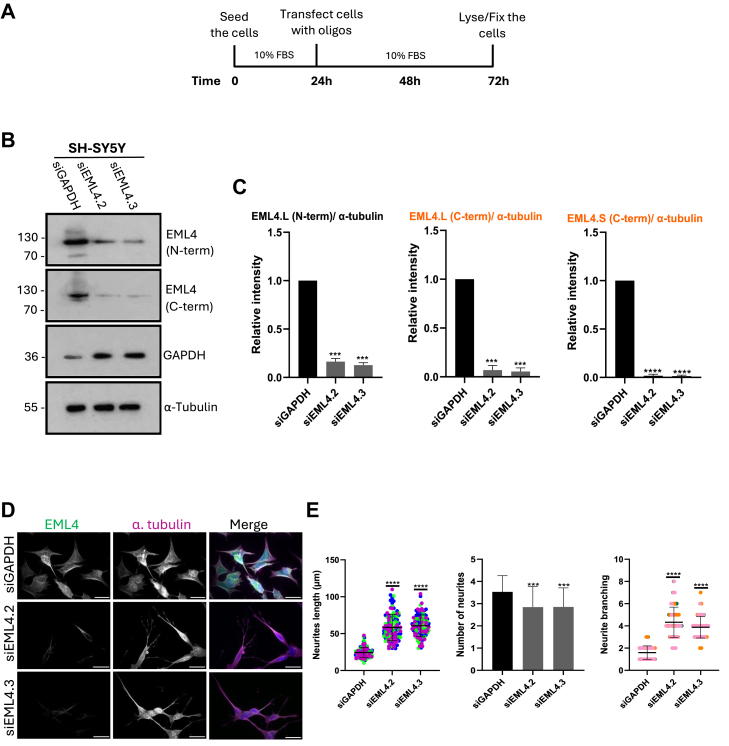
**Depleting EML4 splice variants increases neurite length and branching in undifferentiated SH-SY5Y cells.***A*, the timeline shows the process of depletion of endogenous EML4-L and -S variants in SH-SY5Y cells. *B*, to optimize EML4-L and -S depletion in SH-SY5Y cells, cells were treated with siRNAs4.2 and 4.3 raised against sequences in the C-terminus of the EML4 protein. Cells transfected with siGAPDH were used as controls. Cells were lysed and analyzed by Western blot with the antibodies indicated. The EML4 N-terminal antibody detects the EML4 long variant, whereas the EML4 C-terminal antibody detects both long and short variants. Molecular weights (kDa) are indicated. *C*, the bar charts represent the intensity of EML4 variants normalized to α-tubulin. The bar chart related to the C-term antibody are shown in *orange* colour. Cn = 3, (±S.D.). ∗∗∗*p*-value < 0.001, ∗∗∗∗*p*-value < 0.0001. *D*, SH-SY5Y cells were transfected with the EML4 siRNAs indicated for 48 h before analysis by immunofluorescence microscopy. Cells were stained with EML4 (C-terminal antibody) (*green*), α-tubulin antibodies (red) and DNA with Hoechst 33258 (*blue*) in merge; scale = 20 μm. Zoom scale = 10 μm. *E*, the dot plot reveals the length of neurites after EML4 depletion. The bar chart shows the number of neurites per cell. The second dot plot indicates the neurite branching. This experiment was repeated at least three times, and 20 individual cells were measured in each repeat (±S.D.). *p*-values are indicated.

To deplete EML4 we used two previously designed EML4 siRNAs (siEML4.2 and siEML4.3) that target part of the TAPE domain and are present in both long and short variants. To determine oligo efficiency, SH-SY5Y cells were treated with oligos for 48h, and Western blotting was performed using two EML4 antibodies, one raised against the N-terminus and the other against the C-terminus (Fig. 5, *A* and *B*). The Western blots showed that both EML4 long and short variants were decreased successfully upon treatment with both siRNAs. Reduced expression of the EML4-L was observed with both EML4 antibodies, while loss of the EML4-S was demonstrated in the blot with the EML4 C-terminal antibody. Furthermore, the EML4 N-terminal antibody detected a band of approximately 70 kDa in SH-SY5Y. Interestingly, this band was reduced upon depletion suggesting that this band could indeed be another short splice variant of EML4 (Fig. 5, *B* and *C*).

To test the consequences of EML4 depletion on neurite outgrowth and branching in SH-SY5Y cells, cells were transfected with EML4 siRNAs 4.2 and 4.3 for 48 h. Cells were then analyzed by immunofluorescence microscopy with antibodies against α-tubulin and EML4 (C-terminal antibody) (Fig. 5*D*). Measurements revealed a significant increase in neurite length and branching upon depletion of EML4 with either siRNA in undifferentiated SHSY5Y cells. Consistent with the results obtained with the EML2 depletion, the number of neurites decreased upon EML4 depletion as well (Fig. 5*E*). Hence, we propose that depleting the EML4-L and EML4-S, which act as microtubule stabilizers, leads to microtubule instability, which triggers neurite formation and outgrowth. This suggests that depleting EML4 mimics some of the processes induced by RA addition shown in Figure 3, *D* and *E* rather than activating a different pathway of neurite outgrowth.

## Discussion

Microtubule organization and dynamics are crucial for the development of axons and dendrites during the differentiation of neuronal cells (27, 28). However, we still have much to learn about how microtubule remodeling is regulated during this process. In this study, we looked into how the EML2 and EML4 microtubule-associated proteins might contribute to this. First, we investigated whether EML2 and EML4 undergo alternative splicing during neuronal cell differentiation and how this correlates with microtubule organization and neurite extension. Then we analyzed their localization in undifferentiated and differentiated cells. Finally, we used overexpression and depletion approaches to examine whether long and short splice variants of EML2 and EML4 have contrasting impacts on neuronal cell morphology.

Our results revealed that expression of the EML2-L variant increases while expression of the EML2-S variant is reduced upon differentiation. In contrast, expression of the EML4-L variant decreases while expression of the EML4-S variant remains largely unchanged upon differentiation of human SH-SY5Y neuronal cells. These data are consistent with microarray analysis of cultured mouse hippocampal neurons (21). Although differences in EML splice variant expression were not taken into account in the microarray study, EML2 mRNA was increased, whereas EML4 mRNA was reduced upon differentiation, consistent with our results for EML2 and EML4 long variants. In terms of their localization, the long but not short EML2 variant robustly localized to microtubules before and after differentiation, highlighting the significance of the EML N-terminal region in microtubule binding. Furthermore, EML4-L, which has an intact NTD, associated more strongly with microtubules compared to EML4-S both before and after differentiation.

**Figure 6:**
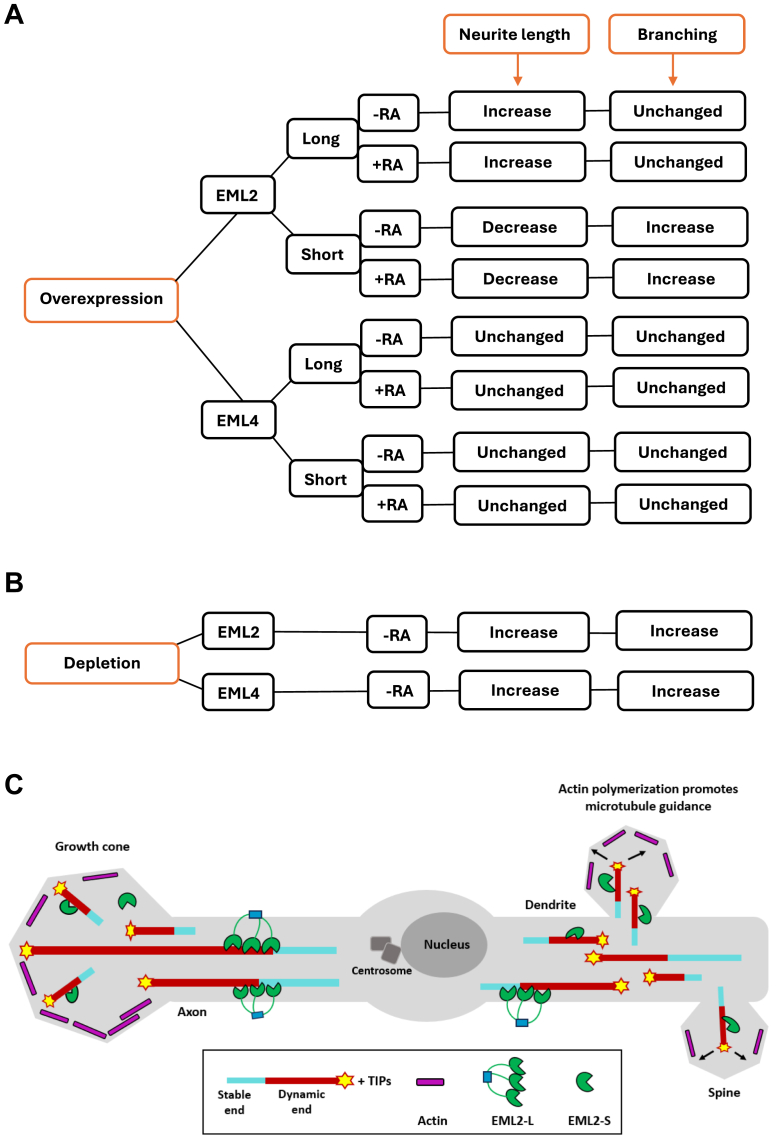
**Summary of the impact of EML2 and EML4 splice variants overexpression and depletion on SH-SY5Y neurite length and branching.***A*, overexpression of EML2-L with or without RA increases the length of neurite and decreases branching while the overexpression of EML2-S decreases the length of neurite and increases branching. Overexpression of both EML4-L and -S does not alter cell morphology with or in the absence of RA. The addition of RA increases the overall length of neurites. *B*, depletion of either EML2 or EML4 increases the length of neurite and branching. *C*, neurite extension happens when EML2-L stabilizes small microtubules in the neurite shaft prior to their entry into the dendritic spine or growth cone. As a result, the membrane is propelled forward, producing longer microtubules that in turn form longer neurites. Meanwhile, EML2-S promotes the formation of numerous short microtubules that can push the membrane in various directions leading to an increase in neurite branching.

EML proteins are thought to be vital for neuronal morphology through their regulation of microtubules. For example, dysfunctions in EMLs, particularly EML1 and EML4, are associated with abnormalities in neuronal morphology (11, 18). A recent study showed that YFP-tagged EML4-L transfected U2OS cells developed extended cytoplasmic protrusions within 24 h, a change reversed by nocodazole, confirming microtubule dependency (29). However, in undifferentiated SH-SY5Y cells, transfection of GFP-tagged EML4-L and -S variants produced no morphological differences after transfection (Fig. 6*A*), possibly due to insufficient incubation time for neurite outgrowth. Furthermore, the stabilization of microtubules leads to a net reduction in the amount of free tubulin. As a result, microtubule-stabilizing proteins can ultimately reduce the rate of microtubule growth (10). It is therefore possible that overexpression of EML4 long and short had no effect on neurite length or cell morphology because of their ability to stabilize microtubules.

In neuronal cells, small microtubules generated through the activity of microtubule-severing proteins act as templates for further microtubule polymerization (30). When these small dynamic microtubules enter dendritic spines or penetrate the growth cone, they produce additional forces that aid the process in which the membrane is pushed forward by F-actin-based rearrangements and polymerization (3). Here, we demonstrated that SH-SY5Y cells transfected with the YFP-tagged EML2-L exhibited longer neurites than YFP-only control cells. Conversely, cells transfected with EML2-S exhibited shorter neurites. Interestingly, these cells exhibited more branches than EML2-L transfected cells (Fig. 6*A*). As microtubule nucleation, stabilization, and development are all necessary for the elongation of axons, we speculate that before entering the dendritic spine or growth cone, EML2-L stabilizes these tiny microtubules in the neurite shaft, which leads to the formation of longer microtubules that produce longer neurites by forcing the membrane forward (Fig. 6*C*). By contrast, EML2-S does not associate with microtubules as it lacks an N-terminal microtubule-binding region. Thus, EML2-S only attaches to monomeric tubulin *via* its TAPE domain, decreasing the amount of free tubulin (10). We hypothesize that this would either generate shorter microtubules or would have no effect on the length of existing short microtubules that enter the dendritic spines or the growth cone. However, EML2-S was reported to bind to depolymerizing microtubules *via* tyrosinated tubulin, leading to rescue of microtubule growth (20). Moreover, dynamic microtubules can form dendritic spines by polymerizing directly into them (31). Hence, we speculate that by generating shorter microtubules and potentially helping them to polymerize, EML2-S may cause the generation of many active microtubules that are ready to push the membrane in various directions, resulting in neurite branching (Fig. 6*C*).

Axon and dendrite nucleation, growth, and elongation, as well as branching, are highly dependent on microtubule organization and dynamics (32, 33, 34). These are tightly regulated by a wide range of microtubule-associated proteins (1), and the data presented in this study demonstrate that this almost certainly includes both EML4 and EML2. Importantly, these proteins undergo alternative splicing, a fundamental process not only for the diversification of protein function but also for brain development (35, 36, 37, 38). Here, we demonstrated that in the absence of EML4 and EML2, neurite lengths are increased in SH-SY5Y cells. However, neurite outgrowth was not the only consequence of depleting EML4 and EML2 long and short variants. Our findings suggested a reduction in the number of neurites as well as a considerable increase in neurite branching (Fig. 6*B*). This was observed in EML2-L depleted cells as well as cells that had both EML2-L and -S depleted simultaneously. Moreover, depletion of both EML4-L and -S variants also led to the same result (Fig. 6*B*). In neuronal cells, microtubule disassembly is crucial for neurogenesis (39). In addition, the stability and assembly of microtubules are regulated by microtubule-associated proteins in axons and dendrites (40). We propose that the microtubule destabilizing activity of the EML2-S variant is required for axon branching and/or remodeling, as it maintains populations of more labile and dynamic microtubules or promotes microtubule severing. Taken together, we speculate that during the early stages of neuronal development, the reduction of EML2-S and EML4-L protein expression leads to neurite outgrowth and branching, whereas an increase in protein expression of EML2-L and EML4-S could promote neurite outgrowth.

These findings have raised a number of questions that will require further studies. For example, the relative expression levels of the different variants need to be assessed in different cell types, with individual studies undertaken on each variant. It will be important to confirm that the short variants are able to interact with soluble tubulin heterodimers, as this is key to potential models for why these should promote microtubule destabilization. Moreover, considering the recent studies from Hotta *et al.* (2022), further investigations are required to determine whether particular tags on the N- or C-termini of EML proteins affect their binding to microtubules and whether EML2-S may indeed specifically bind depolymerizing microtubules, promoting their rescue. Finally, assuming that different EML splice variants have very different functions in neuronal development, it will be important to explore how the alternative splicing of these proteins is regulated.

Overall, our findings highlight how distinct expressions of EML2 and EML4 splice variants promote morphological alterations in SH-SY5Y cells during differentiation. Ultimately, we expect these results to deepen our knowledge of the complex cell biology events taking place in neuronal cells and to provide novel insights relevant to both neurological disorders and cancer.

## Experimental procedures

### Cell culture and transfection

SH-SY5Y cells (human neuroblastoma-derived; subclone of SK-N-SH; ATCC CLR-2266) (41) were cultured in Dulbecco's Modified Eagle Medium: Nutrient Mixture F-12 (DMEM/F12) medium (Invitrogen) supplemented with 10% (v/v) heat-inactivated Fetal bovine serum (FBS), penicillin-streptomycin (100 U/ml and 100 μg/ml, respectively), and 7.5% [w/v] NaHCO_3_. SH-SY5Y cells are neuroblast-like and capable of differentiation into mature neuron-like cells under various conditions, including retinoic acid (RA) treatment. Cells were kept at 37 °C in a humidified atmosphere containing 5% CO_2_ and passaged using trypsin upon reaching ∼80% confluency. Transient transfections were performed upon reaching 60% confluency using the FuGENE transfection reagent (Promega) according to the manufacturer’s instructions. Cells were incubated at 37 °C/5% CO2 for 24 h as required before being processed for Western blot analysis and immunofluorescence microscopy.

### Drug treatment

To induce neuronal differentiation, the density of suspended SH-SY5Y cells was calculated using a Neubauer chamber. Cells were then seeded at a total of 50,000 cells/ml, 24 h before induction. The following day, cells were washed with PBS, and fresh, pre-warmed media with 1% (v/v) FBS containing 10 μM retinoic acid (RA) was added to the cells and incubated for the appropriate time before analysis by Western blot or immunofluorescence microscopy.

### RNAi

Cells were seeded at ∼30% confluency in Opti-MEM Reduced Serum Medium (Invitrogen) supplemented with 10% FBS (no antibiotics). The next day cells were washed and incubated in serum-free Opti-MEM (Gibco) prior to transfection. Transfections were performed using Oligofectamine (Invitrogen) according to the manufacturer’s instructions. siRNA oligos were directed against EML2 (Dharmacon, (HSS119224 (siEML2.1), HSS119225 (siEML2.2), CTM-811663 (siEML2.3), CTM-811664 (siEML2.4), CTM-811665 (siEML2.5)), EML4 (Dharmacon, (HSS120688 (siEML4.2) and Hss178451 (EML4.3) On-Target Plus duplexes) or GAPDH (Ambion, (AM4631)). 4h after the addition of siRNAs, FBS was added to a final concentration of 10% v/v. Cells were incubated with siRNAs for 48h prior to analysis by Western blotting or immunofluorescence microscopy.

### Preparation of cell extracts, SDS-PAGE, and Western blotting

Cells were lysed in RIPA lysis buffer (50 mM Tris-HCl (pH 8.0), 150 mM NaCl, 1% (w/v) SDS, 0.5% (v/v) NP-40/Igepal, 0.5% (w/v) sodium deoxycholate, 0.5% Triton X-100, 1X Protease inhibitor cocktail (Melford Laboratories), 30 μg/ml DNase, 30 μg/ml RNase) on ice for 30 min followed by analysis by SDS-PAGE and Western blotting. Primary antibodies used were against: α-tubulin (1:5000, rabbit antibody, Abcam ab15246), Acetylated tubulin (1:2000, mouse antibody, Sigma T6793, Detyrosinated tubulin (1:5000, rabbit antibody, Millipore AB3201), GAPDH (1:1000, rabbit antibody, Cell Signalling, 2118), EML4(N-term) (1:500, rabbit antibody, Bethyl A301-908A, raised against the N-terminus of the EML4 protein and only detects a long splice variant), EML4(C-term) (1:500, rabbit antibody, Bethyl A301-909A, raised against the C-terminus of the EML4 protein and detects both short and long variants), EML2 (1:500, rabbit antibody, Sigma HPA012757, raised against the C-terminus of the EML2 and detects both a short and long splice variants), EML2 (1:500, rabbit antibody, Proteintech, 13529-1-AP), GFP (1:500, rabbit antibodt, Atlas antibodies, ab6556). Secondary antibodies used were horseradish peroxidase (HRP)-labeled IgGs against rabbit (1:2000, Sigma A6154) and mouse (1:2000, Bethyl A90–116P) antibodies. Enhanced chemiluminescence (ECL) was used to visualize protein bands in accordance with the manufacturer's instructions (Pierce).

### Cloning and Mutagenesis

The University of Leicester's PROTEX cloning service was employed to perform the cloning of constructs into pCEP4, pCDNA3.1, and pLEICS vectors. To generate the EML4-Short variant construct, the Q5 Site-Directed Mutagenesis Kit was used according to the manufacturer’s instructions. The following primer sequences were used: 5′-AAAAGCATAAAACGACCATC-3′ and 5′-AGCAGCAGATGAAAGAGTTTC-3’. To perform PCR, 25 ng of the template DNA plasmid comprising the EML4-Long variant was mixed with the Q5 Hot Start High-Fidelity 2X Master Mix and primers at a final concentration of 10 μM. Following the PCR mutagenesis, 2X KLD reaction buffer and 10X KLD enzyme mix were mixed with the amplified reaction and incubated for 1 h at room temperature. The digested plasmid was transformed into the NEB DH5α competent *E. coli,* which was then plated on selective media and incubated at 37 °C overnight. The following day, colonies were picked individually, the plasmid DNA amplified, and then sent for DNA sequencing.

### Immunofluorescence microscopy

Cells seeded on acid-etched glass coverslips were fixed with ice-cold methanol at −20 °C for at least 30 min. Cells were permeabilized and blocked using 0.2% (v/v) TritonX-100 in (w/v) PBS with 3% BSA in PBS. Primary antibodies used were mouse anti-α-tubulin (1:2000, Sigma T5168), rabbit anti-EML4(N-term) (1:250, Bethyl A301-908A), rabbit anti-EML4(C-term) (1:250, Bethyl A301-909A), rabbit anti-EML2 (1:200, Sigma HPA012757), rabbit anti-EML2 (1:200, Proteintech 13529-1-AP), rabbit anti-GFP (1:1000, Atlas antibodies ab6556). Secondary antibodies were anti-mouse and anti-rabbit IgGs conjugated with Alexa Fluor-488 or Alexa Fluor-594 (1:200, Invitrogen), and DNA was stained with Hoechst 33258. Coverslips were mounted on a glass slide with a drop of mounting solution (80% (v/v) glycerol, 3% (w/v) n-propyl-gallate in PBS). The edges of the coverslips were sealed by a clear nail varnish and kept in the dark at 4 °C. The VisiTech Infinity three confocal microscope equipped with a Hamamatsu C11440 -22CU Flash 4.0 V2 sCMOS camera and a Plan Apo VC 60x (NA 1.4) or Plan Apo 100 x objective (NA 1.47) was used to acquire Z-stacks with a step size of 250 nm. The images were further analyzed using Fiji (42).

### Quantification of protein co-localization

Immunofluorescence microscopy (see above) was used to evaluate fixed cells labeled with GFP/EML4/EML2 and α-tubulin antibodies, and co-localization was calculated using Fiji. A line of 2.5 μm in length was drawn across several microtubule strands and the Plot-multicolor 4.3 macro (https://github.com/KeesStraatman/Multi-color-line-profile-plot) was used to measure the intensity along the line of the two different antibody signals and the Pearson's correlation coefficient was calculated from this (43). Pearson's correlation coefficient was determined by assessing five distinct lines of 10 cells per experiment, and each experiment was performed in triplicate. An R-value of greater than 0.5 is generally considered a strong association.

### Analysis of neurite length, number, and branching

Cells were stained with α-tubulin (microtubules) and Hoescht-33528 (DNA), and images were analyzed using Fiji. Microtubule and DNA images were merged, and specific parameters were measured: (i) neurite length – each cell's longest neurite was selected, and its length from the nucleus to the tip of the neurite was measured; (ii) number of neurites – the number of protrusions from the cell body was counted; (iii) neurite branching – the number of extensions from each individual protrusion was counted.

### Statistical analysis

To determine the statistical significance of data, GraphPad Prism Version 10 was used to calculate the average of at least three independent experiments (each color represents one repeat) together with means and standard deviations. Student’s unpaired two-tailed *t* test, assuming unequal standard variation, was used to compare datasets from two groups, whereas one-way ANOVA with *post hoc* tests was used to compare datasets from more than two groups. *p*-values represent: ∗*p* < 0.05, ∗∗*p* < 0.01, ∗∗∗*p* < 0.001, ∗∗∗∗*p* < 0.0001; ns, nonsignificant.

## Data availability

All data described are contained within this article or the supporting information.

## Supporting information

This article contains supporting information.

## Conflict of interest

The authors declare that they have no conflicts of interest with the contents of this article.
