# Supplementary material for: EML2 and EML4 splice variants regulate microtubule remodeling during neuronal cell differentiation
Source: J Biol Chem. 2025 May 19;301(6):110252. doi: 10.1016/j.jbc.2025.110252 (PMC12182296; doi:10.1016/j.jbc.2025.110252)

SUPPLEMENTARY FIGURES

Figure S1. Retinoic acid induces neurite growth in SH-SY5Y cells. (A) Light microscopy shows the differences in neurite lengths in SH-SY5Y cells that were treated without (undifferentiated, left panel) or with (differentiated) retinoic acid for 10 days (D10). (B) The bar chart represents the neurite length in cells treated with and without 10 µM retinoic acid (+ and – RA) for 10 days. The data represents mean neurite length from at least 30 cells (±S.D.). $p < 0.0001$.

Figure S2. Expression of acetylated and detyrosinated tubulins remained relatively unchanged during differentiation. (A) Lysates were prepared from SH-SY5Y human neuronal cells at the indicated days (D0 to D20) after the addition of RA to induce differentiation. Western Blots were undertaken with acetylated tubulin (Sigma) and detyrosinated tubulin (Millipore) to check expressions of these proteins at different phases of differentiation. The α-tubulin antibody was used as a loading control. Molecular weights (kDa) are indicated on the left. (B) Bar charts represent the intensity of acetylated, and detyrosinated tubulin across the 20 days of induction with RA, normalized to α-tubulin. Data obtained from three repeats, n=3 (±S.D.).

Figure S3. Overexpression of EML2 short variant reduces neurite length in differentiated SH-SY5Y cells (A) The timeline shows the experimental process in which SH-SY5Y cells were first subjected to 96 h RA-induced differentiation and then transfected with YFP only, YFP.EML2-L and -S. (B) Cells were differentiated for 96 hours and then transfected with EML2 long and short variants before they were fixed and stained with α-tubulin (red) and GFP (green) antibodies. DNA was stained with Hoechst 33258 (blue in merge). Scale bar, 10 µm. (C) Dot plots indicate the length of neurite in SH-SY5Y cells differentiated for 96 h (four days, D4) then were transfected with EML2 long and short variants. The experiment was performed at least three times and the length of neurites in 14 cells counted in each experiment (±S.D.). * p-value: 0.0373 and *** p-value < 0.001.

A

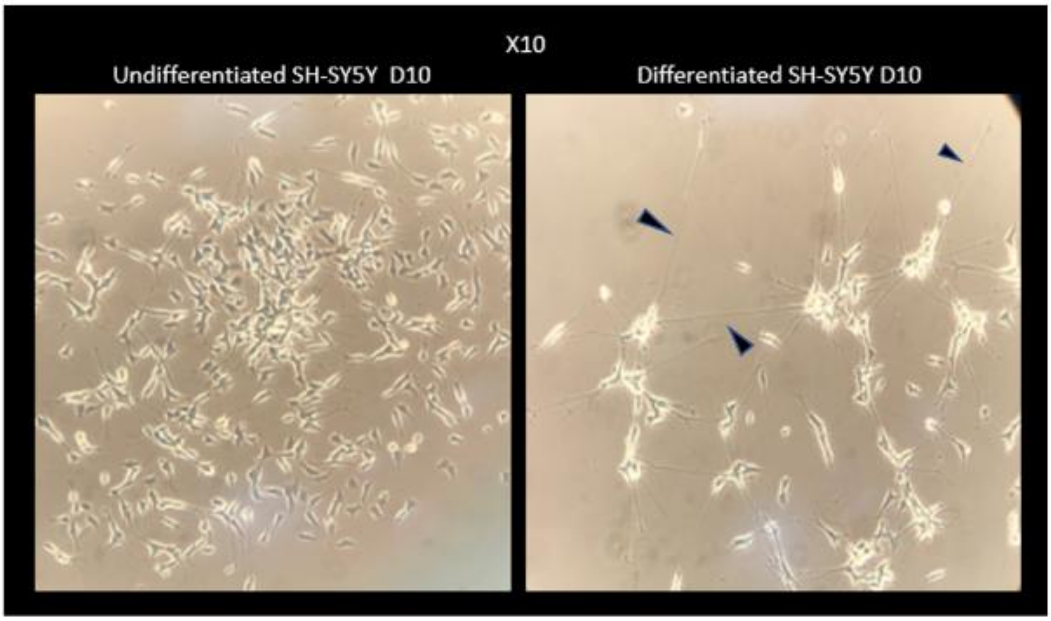
X10
Undifferentiated SH-SY5Y D10
Differentiated SH-SY5Y D10

B

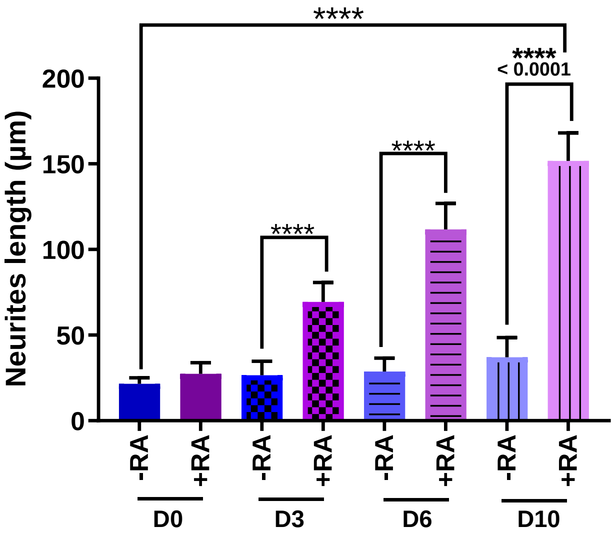
Neurites length (µm)
200
150
100
50
0
****
****
< 0.0001
-RA
+RA
D0
D3
D6
D10

A

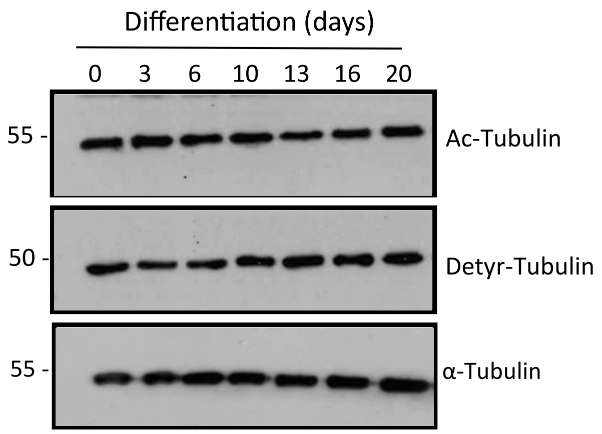
Differentiation (days)
0 3 6 10 13 16 20
55 -
Ac-Tubulin
50 -
Detyr-Tubulin
55 -
α-Tubulin

B

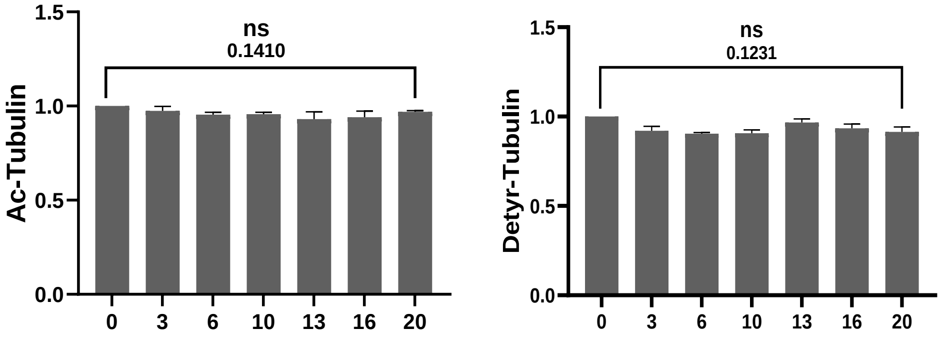
ns
0.1410
Ac-Tubulin
1.5
1.0
0.5
0.0
0 3 6 10 13 16 20
ns
0.1231
Detyr-Tubulin
1.5
1.0
0.5
0.0
0 3 6 10 13 16 20

Marasi *et al.* (2025) Figure S3

A

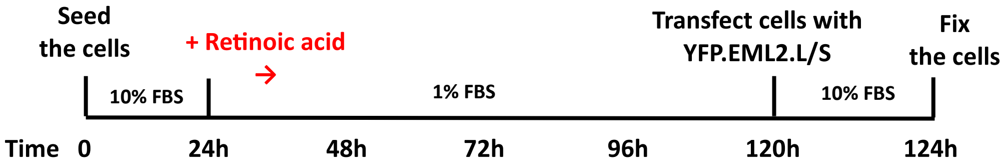

B

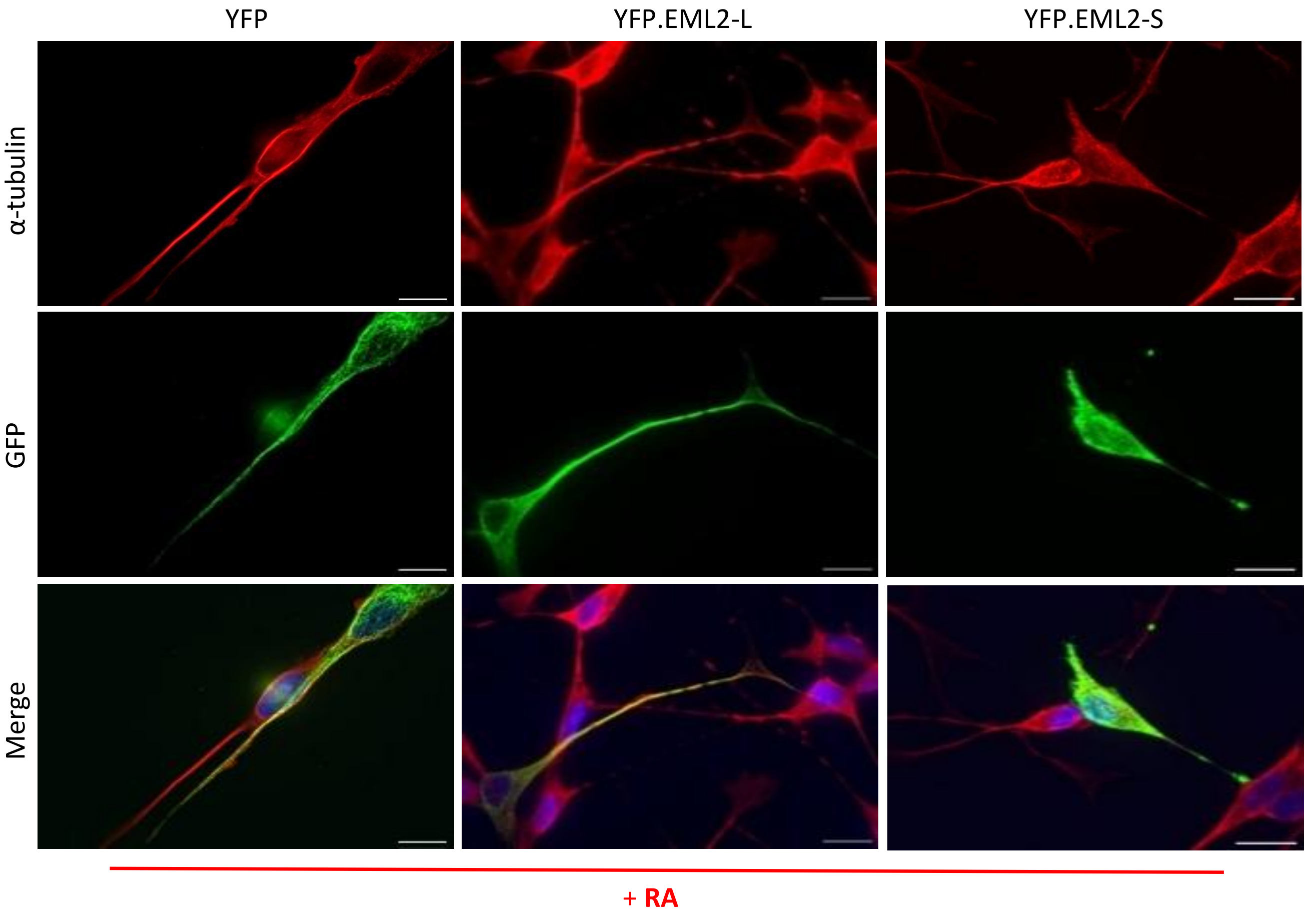

C

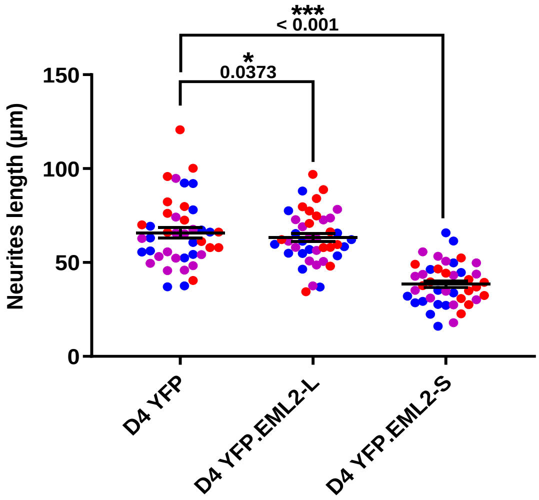

Supplement: Supplementary Data [file mmc1.pdf]
